# Generative AI unlocks PET insights: brain amyloid dynamics and quantification

**DOI:** 10.3389/fnagi.2024.1410844

**Published:** 2024-06-17

**Authors:** Matías Nicolás Bossa, Akshaya Ganesh Nakshathri, Abel Díaz Berenguer, Hichem Sahli

**Affiliations:** ^1^Department of Electronics and Informatics (ETRO), Vrije Universiteit Brussel (VUB), Brussels, Belgium; ^2^Interuniversity Microelectronics Centre (IMEC), Leuven, Belgium

**Keywords:** GAN, ODE, AD progression model, PET, amyloid, brain, ADNI

## Abstract

**Introduction:**

Studying the spatiotemporal patterns of amyloid accumulation in the brain over time is crucial in understanding Alzheimer's disease (AD). Positron Emission Tomography (PET) imaging plays a pivotal role because it allows for the visualization and quantification of abnormal amyloid beta (Aβ) load in the living brain, providing a powerful tool for tracking disease progression and evaluating the efficacy of anti-amyloid therapies. Generative artificial intelligence (AI) can learn complex data distributions and generate realistic synthetic images. In this study, we demonstrate for the first time the potential of Generative Adversarial Networks (GANs) to build a low-dimensional representation space that effectively describes brain amyloid load and its dynamics.

**Methods:**

Using a cohort of 1,259 subjects with AV45 PET images from the Alzheimer's Disease Neuroimaging Initiative (ADNI), we develop a 3D GAN model to project images into a latent representation space and generate back synthetic images. Then, we build a progression model on the representation space based on non-parametric ordinary differential equations to study brain amyloid evolution.

**Results:**

We found that global SUVR can be accurately predicted with a linear regression model only from the latent representation space (*RMSE* = 0.08 ± 0.01). We generated synthetic PET trajectories and illustrated predicted Aβ change in four years compared with actual progression

**Discussion:**

Generative AI can generate rich representations for statistical prediction and progression modeling and simulate evolution in synthetic patients, providing an invaluable tool for understanding AD, assisting in diagnosis, and designing clinical trials. The aim of this study was to illustrate the huge potential that generative AI has in brain amyloid imaging and to encourage its advancement by providing use cases and ideas for future research tracks.

## 1 Introduction

The discovery of the presence of Amyloid-beta (Aβ) plaques in the brain was a crucial breakthrough in understanding the pathology of Alzheimer's disease (AD). The development of Positron Emission Tomography (PET) imaging has allowed for the visualization of Aβ plaques *in vivo*, providing unprecedented insights into the progression of AD (Klunk et al., 2004). Since then, many efforts have been made to study the regional and temporal patterns of amyloid accumulation and identify the stages of its spread throughout the brain (Thal et al., 2002; Murray et al., 2015). Noteworthy, the findings are not always consistent, and different amyloid staging models were proposed (Grothe et al., 2017, cf. Koychev et al., 2020). More accurate and versatile tools to analyze PET images and evaluate the amyloid pathology progression could help unravel the source of these discrepancies.

Numerous methodologies for amyloid quantification have been introduced over time. In general, Magnetic Resonance Imaging (MRI) scans have been crucial in accurately mapping the accumulation of amyloid in the brain because they provide a detailed image of the brain's structure. This is usually followed by brain parcellation and mapping of brain segmentation to the PET image. After that, a group comparison of normalized PET signals within each region is carried out (Grothe et al., 2017; Moffat et al., 2022; Patow et al., 2023). These techniques, however, are often hindered by complex preprocessing steps and the requirement for an accompanying MRI scan (Pemberton et al., 2022). In addition, different preprocessing pipelines produce different estimation results (Kolinger et al., 2021). To overcome these limitations, some researchers have recently suggested automatic quantification methodologies based on deep learning solely utilizing amyloid PET images (Kim et al., 2019; Reith et al., 2020; Komori et al., 2022; Lee et al., 2022; Kang et al., 2023; Maddury and Desai, 2023). These networks are specifically trained to estimate the cortical-to-cerebellum standardized uptake value ratio (SUVR) average, determine amyloid positivity, or compute a few regional SUVR averages. Consequently, the rich spatial information inherent in the data is lost.

An alternative approach, embraced in this work, is to encode PET images in a convenient space that can be used later for several analyses. Combining this representation with a generative process provides a powerful modeling framework for analysis and simulation (Schön et al., 2022). Generative artificial intelligence (AI) makes use of advanced models like generative adversarial networks (GANs), diffusion models, and variational autoencoders (VAEs) to learn complex data distributions and generate realistic synthetic data (Alamir and Alghamdi, 2022). These models have shown their potential to mitigate the issue of data scarcity, improving algorithm performances by artificially expanding the pool of images available for training (Frid-Adar et al., 2018; Yi et al., 2019). They were also integrated into AI interpretability frameworks through counterfactual examples to understand how AI systems make decisions or identify the source of errors they produce (Chang et al., 2021).

Progression models take image generation a step further by generating synthetic images and simulating the evolution of a synthetic patient over time. These models can provide a continuous, patient-specific timeline of disease progression, enhancing our understanding of disease trajectories and the factors influencing them (Ravi et al., 2022). In the context of clinical trial design, longitudinal models allow for the measurement of expected pathological changes from the generated images, providing valuable insights into disease progression. Data augmentation via simulation can be used to optimize different aspects of the trial, such as sample sizes, enrollment criteria, trial duration, and the number of intermediate time points. This is especially important when conducting PET imaging studies due to the technique's high cost, technical limitations, and invasive nature.

Generative adversarial networks (GANs) (Goodfellow et al., 2014) synthesize realistic-looking features by learning the sample distribution from real data, providing a powerful way to model and manipulate images directly. In neuroimaging, the latent space of GAN models was used as a low-dimensional representation for uncovering disease-related imaging patterns (Wang et al., 2023). Several works studied progression due to age or disease. Bowles et al. (2018) built a progression model for AD and a latent encoding with disentangled AD features using a Wasserstein GAN (W-GAN) (Arjovsky et al., 2017). MRI scans with different grades of AD severity were simulated by manipulating specific elements of the latent encoding. A GAN variant was used to identify subtypes of Alzheimer's disease in MRI scans by studying the pathologic neuroanatomical heterogeneity in brains with AD and the differences with healthy brains (Yang et al., 2021). The disease progression was described as the hypothetical order in which subtypes are visited. In Ravi et al. (2022), a 2D plus time model was designed to generate high-resolution, longitudinal MRI scans that mimic subject-specific neurodegeneration in aging and dementia. It proposes a modular framework based on adversarial training and spatiotemporal, biologically-informed constraints. The progression model is based on a Conditional Deep Autoencoder (CDA) (Zhang et al., 2017). In Zhao et al. (2021), a GAN model based on 3D U-Net was proposed to generate MRI images at pre-specified future time-points, 1 and 4 years after the baseline, conditioned on the image at the baseline and other covariates. A similar approach was applied in Campello et al. (2022), where a conditional GAN was used to synthesize older and younger versions of a heart MRI scan using only cross-sectional data. Other works used GANs for disease prediction or classification without modeling AD evolution. In Gao et al. (2022) and Pan et al. (2022), GAN-based models were used to impute missing PET images from the available MRI images. This approach allowed them to utilize the complete multimodal data (both MRI and PET images) for more accurate disease classification.

In this study, we propose to leverage a 3D GAN model, 3D-StyleGAN (Hong et al., 2021), for PET image representation and synthesis. 3D-StyleGAN is an extension of StyleGAN (Karras et al., 2019) developed for medical images and evaluated on MRI images. StyleGAN-based architectures generate highly realistic and diverse images. They also contain an intermediate latent space less entangled than the input space, showing better control, and interpretability on image synthesis compared to previous generative models (Karras et al., 2018; Hong et al., 2021). We quantify the latent space information content by fitting a linear regression model to predict SUVR and evaluate the performance degradation when projecting the latent vector in even lower dimensional spaces given by Principal Component Analysis. To illustrate the potential of this representation for modeling brain amyloid evolution, we use a non-parametric ODE model based on Gaussian Process (GP) regression (Bossa et al., 2022) to model amyloid dynamics on a five-dimensional PCA subspace of the latent space. We visualize the individual evolutions on this subspace and generate synthetic image trajectories for a few examples to visually compare synthetic and real images after four years of evolution.

Our approach presents several differences from the previous works. First, it is the first to study the amyloid progression in PET images. Previous studies used generative AI to model progression only MRI images and, therefore, the neuroanatomical changes. Second, our approach is more straightforward and versatile because several generative models can be combined with different progression models. Previous works used conditional networks to learn progression, which requires a large amount of longitudinal data. Compared with the conditional networks, our framework allows recomputing the dynamics in new settings, diseases, or sub-populations or incorporating more covariates without re-training the GAN model, which could be pre-trained on large cross-section databases.

## 2 Materials and methods

### 2.1 Participants

We used publicly available data from the Alzheimer's Disease Neuroimaging Initiative (ADNI) database^1^ to fit the models. The ADNI was launched in 2003 as a public-private partnership, led by Principal Investigator Michael W. Weiner, MD. The primary goal of ADNI has been to test whether serial magnetic resonance imaging (MRI), positron emission tomography (PET), other biological markers, and clinical and neuropsychological assessment can be combined to measure the progression of mild cognitive impairment (MCI) and early Alzheimer's disease (AD). All ADNI participants provided written informed consent, and study protocols were approved by each local site's institutional review board. All methods were carried out in accordance with relevant guidelines and regulations. Further information about ADNI, including full study protocols, complete inclusion and exclusion criteria, and data collection and availability can be found at adni.loni.usc.edu.

The amyloid PET scans are from the longitudinal PET imaging study conducted during the ADNI-2 and ADNI-GO phase. These PET images are acquired by administering ^18^*F* Florbetapir (^18^*F* AV-45) to the subject and are imaged 50–70 min post injection for continuous 20 minutes to obtain the brain PET images^2^. All subjects with AV45 PET scans were included, resulting in a total of 1,259 participants and 2,920 AV45 PET images.

The characteristics of the population included in the analysis are summarized in Table 1. The mean number of PET scans per subject was 2.3, with 800 participants having at least two scans and 243 having four scans. The mean PET follow-up duration was 2.6 years, with 280 participants with more than 5 years of follow-up.

**Table 1:** Demographics for included participants.

	**CN**		**MCI**		**AD**	
	**Male**	**Female**	**Male**	**Female**	**Male**	**Female**
Participants (*N*)	222	216	345	260	127	89
*N* APOE-ϵ4 (0/1/2)	159/51/5	139/58/9	170/123/33	137/90/22	47/46/27	25/45/14
PET scans (*N*)	623	577	749	532	244	195
Follow-up (years)	3.0 (7.5)	2.6 (7.3)	2.0 (5.9)	1.8 (5.6)	0.5 (2.0)	0.7 (2.1)
Follow-up ($\frac{N\;\text{scans}}{N\;\text{participants}}$)	2.6 (5)	2.4 (4)	2.0 (4)	1.9 (3)	1.3 (2)	1.4 (2)

APOE information was not available for all of them. Follow-up is reported as: *mean* (90*th* percentile).

### 2.2 Image preprocessing

PET scans consist of four 5-min frames co-registered to the base frame and averaged into a single static frame. Each scan is then reoriented into a standard 160 × 160 × 96 voxel image grid, having 1.5 mm cubic voxels (Jagust et al., 2015). Since these scans are procured from different scanners of varied resolutions, the image sets are filtered with a scanner-specific filter function in order to have uniform resolution across all images.^3^

SUVR estimates for most of these scans are provided with the ADNI dataset and were estimated using various image preprocessing steps. This includes the coregistration of a PET image of each subject to the MRI scan of that subject using SPM software.^4^ Further, Freesurfer software^5^ is used to skull-strip, segment, and delineate cortical and subcortical regions in all MRI scans. SUVR is thus calculated by dividing the conventional average across the four main cortical regions (frontal, anterior/posterior cingulate, lateral parietal, lateral temporal) by one of the reference regions.^6^

### 2.3 GAN-based latent space representation

GAN is a deep learning-based generative modeling approach in which two networks are trained simultaneously in a min-max game via an adversarial process (Goodfellow et al., 2014). A GAN architecture involves two networks: a generator and a discriminator model. The generator model captures the data distribution to generate new plausible data samples. The discriminator model estimates the probability that a sample came from the training data rather than the generator. The generator network *G* takes a normally distributed random vector z~N(0,I) as input, which is used to seed the generative process. This noise vector is transformed to generate samples *G*(**z**) from the data distribution. Discriminator network *D* gets real data or generator output *G*(**z**) as inputs, one at a time. The discriminator network is trained to distinguish these inputs as real or fake. The generator network, in turn, is trained to fool the discriminator into accepting its outputs as being real. During training, the generator tries to minimize the loss while the discriminator tries to maximize it.

#### 2.3.1 3D-StyleGAN

Various modifications and innovations have been developed using this original framework. In the original generator network, the latent vector **z** is provided to the generator through an input layer. The input latent space *Z* is usually entangled and challenging to interpret and manipulate. StyleGAN (Karras et al., 2018, 2019) is a family of GANs that use an alternative generator architecture borrowed from style transfer architecture (Huang and Belongie, 2017). They include a disentangled latent space to generate highly realistic images with control over the image synthesis process via style mixing.

**Figure 1:**
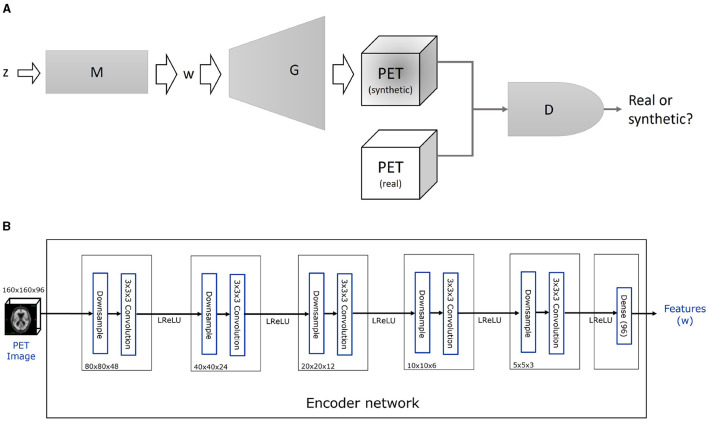
Components of the proposed methodology. **(A)** Schematic representation of 3D-StyleGAN. A mapping network M maps a normally distributed random vector *z* to an intermediate latent vector *w*, which is then used to feed a style-based generator network *G*. The generator *G* produces a realistic-looking synthetic PET image. A discriminator network *D* is trained to differentiate the synthetic from the real images while *D* and *M* are trained adversarially, i.e., to prevent the discriminator from detecting the synthetic image. The details of each block's architecture and training are given in Section 2.3 and in Hong et al. (2021). **(B)** Details of the encoder network, *E*, used to invert the pre-trained 3D-StyleGAN generator, *G*. Five convolutional layers followed by a dense layer map a PET image into the intermediate latent vector *w* = *E*(*PET*). A mean squared error loss on the difference between the input and reconstructed images ||*PET* − *G*(*E*(*PET*))||^2^ is used to train the encoder. **(A)** 3D-StyleGAN. **(B)** Encoder.

The main components of StyleGAN are illustrated in Figure 1A. The input vector **z** is embedded into an intermediate latent space *W* through an 8-layer fully connected (FC) MLP mapping network *M*, i.e., **w** = *M*(**z**). Another particularity of StyleGAN is in its generator or synthesis network *G*. A constant layer is used as input to a set of successive convolutional networks, each modulated by the latent vector **w** via respective affine transforms *A*. This leads to a less entangled, more linear representation of latent factors of variation (Karras et al., 2018). Later, the StyleGAN generator was re-designed to improve image quality and remove artifacts, leading to StyleGAN2 (Karras et al., 2019).

StyleGAN and StyleGAN2 were initially developed to synthesize high-quality 2D images and gained popularity for generating realistic human faces. More recently, StyleGAN2 was extended to three-dimensional (3D) medical image synthesis. 3D-StyleGAN is an adaptation of StyleGAN2 to enable synthesis of 3D medical images (Hong et al., 2021). Several changes are made to the original StyleGAN2 architecture, including the depths of the filter maps and latent vector sizes, which are significantly reduced to limit the high memory requirements and computational complexity. See Hong et al. (2021) for additional implementation details.

#### 2.3.2 Encoder

An encoder *E* is added to invert the synthesis network of the 3D-StyleGAN to obtain the intermediate latent vector representation of the real images. The task of the encoder network is to map the input PET image to the output features **w** as illustrated in Figure 1B. Each block in this network consists of filtered downsampling followed by a 3 × 3 × 3 convolution layer. Leaky Rectified Linear Unit (LReLU) activation is applied after each block. No output activation is applied after the dense layer. These convolution layers have a filter depth of 16, 32, 32, 32, and 16. The size of the final dense layer of the network is 96 to match the intermediate latent vector size of the 3D-StyleGAN generator.

### 2.4 PET image trajectory modeling using non-parametric ODEs

We follow the approach proposed in Bossa et al. (2022) to estimate the brain amyloid evolution. Feature velocities are modeled as a function of their current value and, eventually, other covariates. This is expressed mathematically as a set of ordinary differential equations (ODEs):


(1) $$\begin{array}{c} d{\text{x}}_{i}\left(t\right)/dt=\text{V}\left({\text{x}}_{i}\left(t\right),{\text{c}}_{i}\right)\;, \end{array}$$

where **x**_*i*_(*t*) is a dynamical feature vector of subject *i* at time *t*, **c**_*i*_ is a vector with covariates (age and number of APOE−ϵ4 alleles in our case), and **V**(·, ·) is a vector function to be estimated from the data. We opted to model **V**(·, ·) using Gaussian process (GP) regression, as described in Bossa et al. (2022). Observed velocities were approximated with finite differences between features from consecutive images of the same subject. GP regression was used to estimate a smooth function for each component of the velocity field.

Three assumptions motivate this choice:

1. The velocity observations are noisy due to the finite difference approximation, intersubject variability that can not be modeled with covariates, and feature variations unrelated to amyloid progression.
2. The velocity is not expected to change abruptly between two similar PET images. This is grounded in biological considerations and the observed patterns and rates of amyloid accumulation.
3. Velocity is only determined by current status and covariates. This is the most limiting assumption because it rules out the possibility that two individuals have the same condition and, at some point, they start to diverge in their evolution due to unknown factors, such as genetic or environmental differences. However, accounting for these possibilities would present significant challenges requiring more training data and complex modeling tools.

After learning the velocity model, it can predict the velocity for any given combination of latent representation and covariates. We forecast long-term trajectories from the given initial states using forward Euler integration.

The feature vector **x** could be either the latent vector **w** or a low-dimensional representation of it. Further reducing the dimensionality may increase the robustness of the dynamical pattern estimation and reduce the risk of overfitting. We used Principal Component Analysis (PCA) to reduce the dimensionality and to investigate if the information in the latent space *W* is redundant for amyloid quantification and progression modeling. Let ${\left\{{\text{v}}_{k}\right\}}_{k=1}^{K}$ be the first *K* Principal Components (PCs), and **b**^*i*^ the corresponding vector of principal scores for subject *i*, such that ${\text{w}}^{i}\approx \bar{\text{w}}+\overset{K}{\underset{k=1}{\sum }}{\text{v}}_{k}b_{k}^{i}$, where $\bar{\text{w}}$ is the latent vector mean across subjects.

Let ${Î}_{t}^{i}$ be a synthetic PET image trajectory whose initial value corresponds to the baseline PET image of the participant *i*, $I_{\text{baseline}}^{i}$, so that ${Î}_{0}^{i}=G\left({\text{w}}_{0}^{i}\right)\approx I_{\text{baseline}}^{i}$ with ${\text{w}}_{0}^{i}=E\left(I_{\text{baseline}}^{i}\right)$. Then, ${Î}_{t}^{i}$ is computed as


(2) $$\begin{array}{c} {Î}_{t}^{i}=G\left(\bar{\text{w}}+\overset{K}{\underset{k=1}{\sum }}{\text{v}}_{k}PM_{k}\left({\text{b}}_{0}^{i},{\text{c}}^{i},t\right)\right) \end{array}$$

where *PM*_*k*_(**b**_0_, **c**, *t*) is the output of the progression model for component *k*, i.e., the result of integrating the velocity field **V**(**b**(τ), **c**) starting at **b**(0) = **b**_0_ during a time τ = *t*.

The velocity field **V**(**b**(τ), **c**) is computed as follows. Let *B* denote the space of principal scores, i.e., **b**_*j*_ ∈ *B*. A set of velocities in *B* is computed using finite differences of principal scores from consecutive images of the same subject. A Gaussian Process (GP) regression is then fitted to these velocity estimations, resulting in a smooth velocity field in the *B* space.

**Figure 2:**
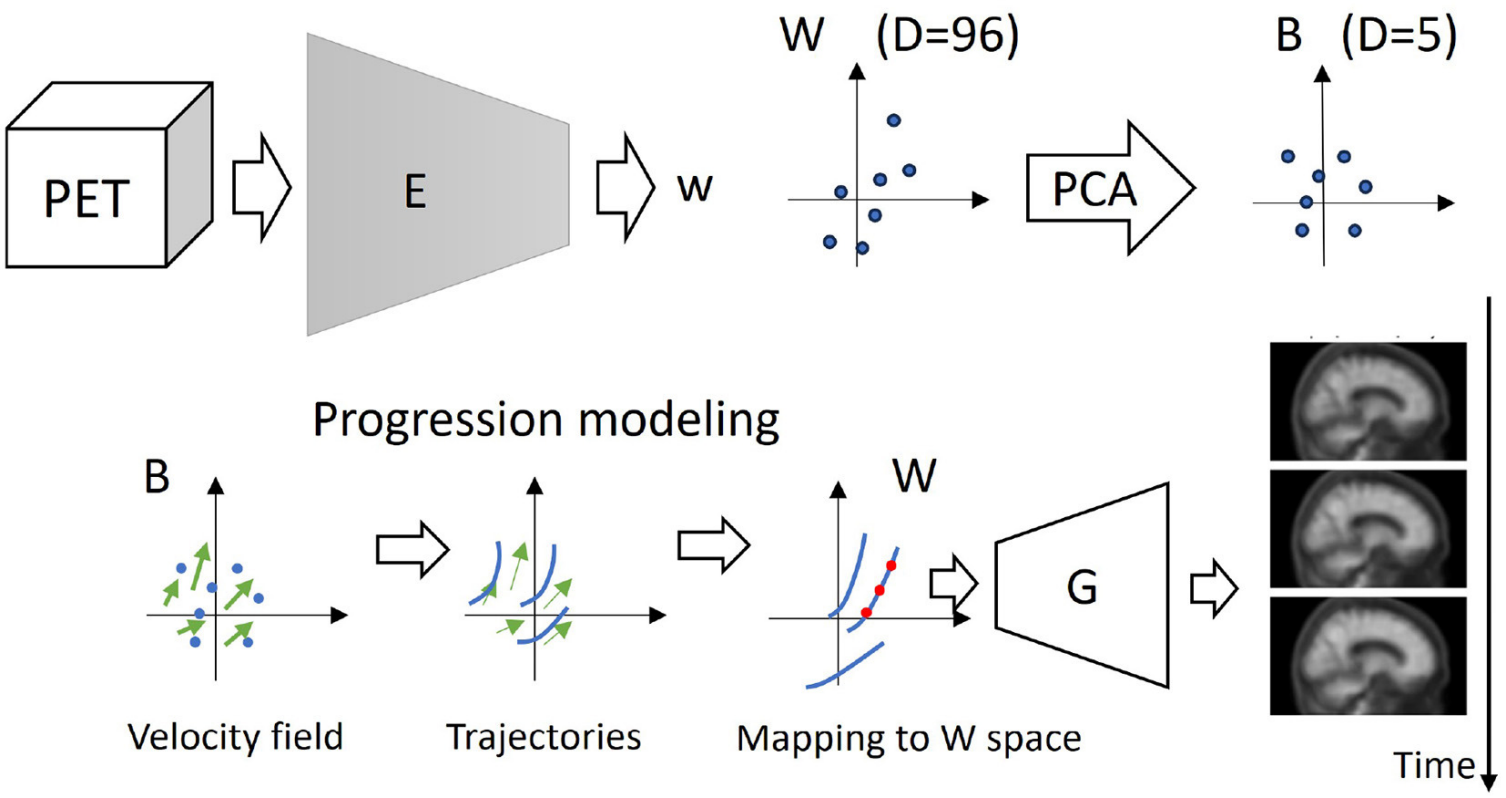
Amyloid feature representation and progression modeling pipeline. **Top** (from left to right): The encoder (*E*, see Figure 1) projects each PET image into the 3D-StyleGAN latent representation *W*. Dimensionality is further reduced using PCA. **Bottom** (from left to right): A smooth, continuous velocity field is estimated from the participants' projected PET image pairs. The velocity field is used to predict trajectories on the PCA subspace, which are then projected back to the 3D-StyleGAN latent space *W*. The 3D-StyleGAN synthesis network (*G*, see Figure 1) generates a synthetic PET image evolution of a given synthetic or real patient.

We explored the performance of the PCA projected latent vector to predict SUVR for different numbers of components. Based on this analysis, we selected the optimal number of PCA dimensions and fitted the progression model to this subspace. Then, we projected back the estimated trajectories to the corresponding hyperplane on the original latent space *W* and generated the images. The entire pipeline is illustrated in Figure 2.

## 3 Results

### 3.1 Latent space representation

#### 3.1.1 SUVR prediction

**Figure 3:**
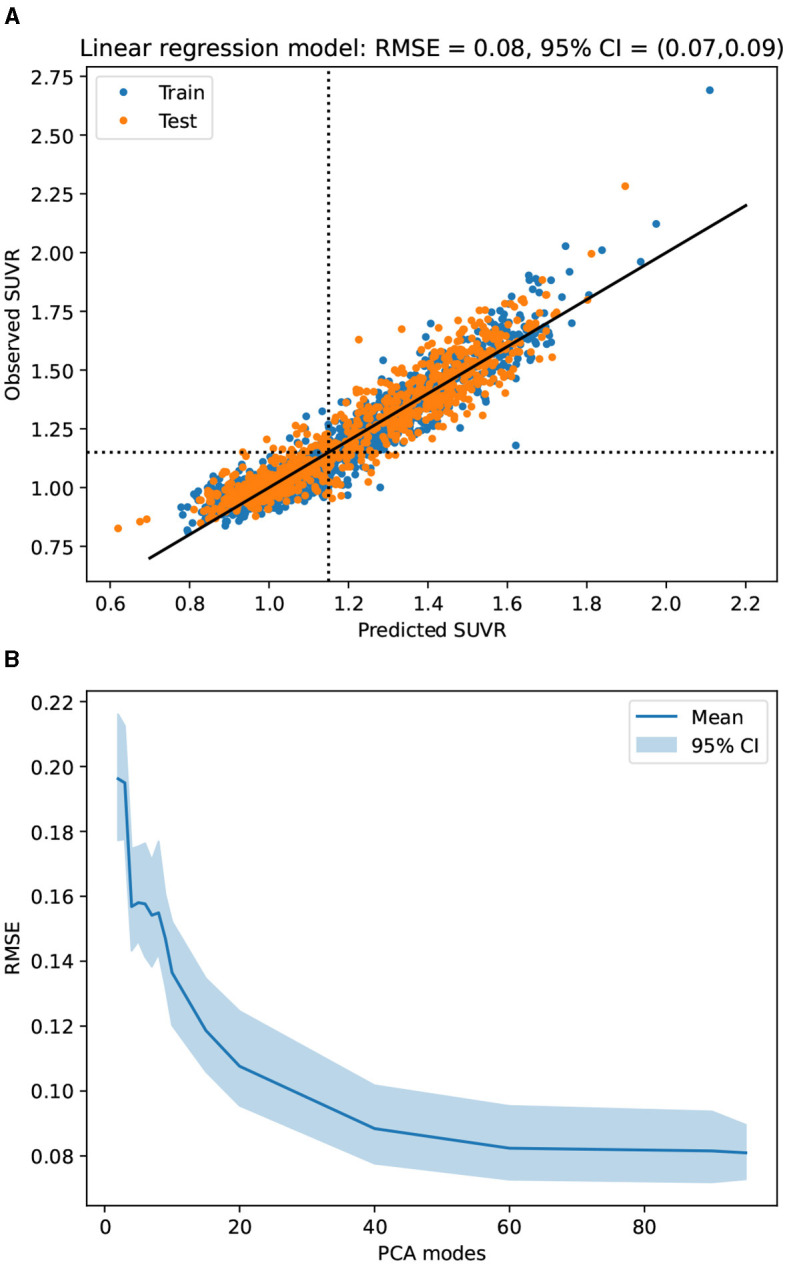
Performance (RMSE) of a linear regression model trained to predict SUVR from the 3D-StyleGAN latent representation **(A)** and the PCA projections **(B)**. Confidence intervals (CI) were estimated with 100 random training/test splittings. **(A)** Observed vs predicted SUVR in train and testing sets (random partition 65%/35%). **(B)** SUVR prediction error vs number of PCs.

We first aim at determining the quality and redundancy of the information contained in the latent representation. For that, trained a linear regression model to predict SUVR solely based on the complete latent vector. Figure 3A shows the observed vs. predicted SUVR for the training and test sets. We made a bootstrap experiment with 1,000 random training/test splitting and obtained (*mean* (95*%CI*)) *RMSE* = 0.081(0.072, 0.092), *AUROC* = 0.985(0.975, 0.992), and *MAE* = 0.061(0.055, 0.067). This performance is comparable to that of DL models specifically trained for predicting SUVR (Kim et al., 2019; Reith et al., 2020; Komori et al., 2022; Lee et al., 2022; Kang et al., 2023; Maddury and Desai, 2023).

Then, we did the same bootstrap experiment on the PC projection for several number of PCA dimensions (see Figure 3B). There is a dramatic improvement in SUVR prediction around five PCs, followed by a plateau (see Figure 3Bright panel). Performance improves again after seven PCs, but no other clear-cut points exist. Therefore, at five dimensions, it is the first and most evident “elbow” in the SUVR error prediction curve.

#### 3.1.2 Progression modeling

We explored the amyloid dynamics in a low-dimensional PCA subspace. We kept the first five PCs for the qualitative analysis for two reasons. First, visualization is more complex and prone to arbitrary choices on higher dimensions. Second, from the previous analysis (see Figure 3), we concluded that five PCs is the smaller number of PCA dimensions containing a sufficient amount of amyloid-related information.

**Figure 4:**
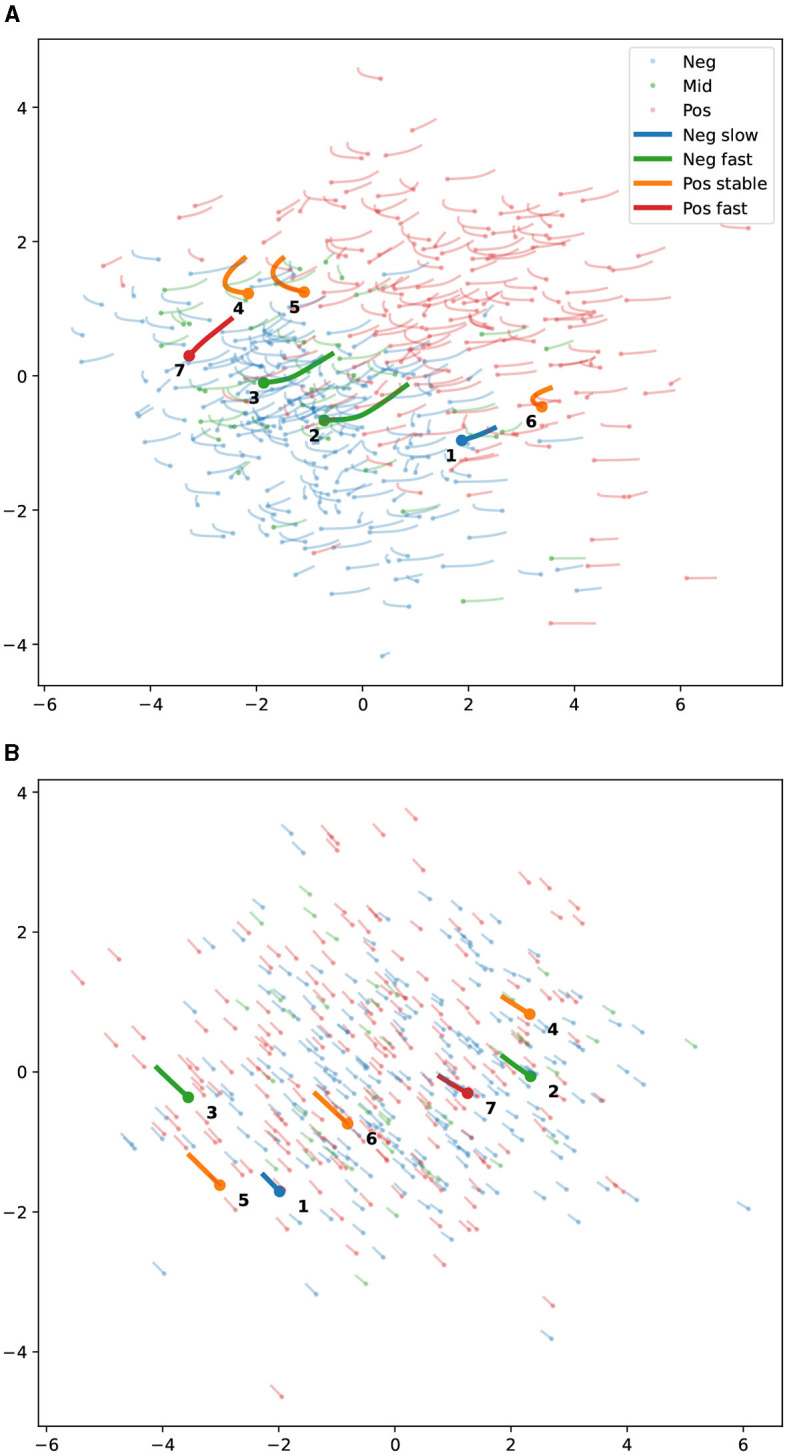
Amyloid PET dynamics on a PCA subspace of the 3D-StyleGAN latent representation space. Thin semitransparent lines represent five years of evolution, with the dots being the starting points. The colors represent the amyloid positiveness according to the SUVR: blue for amyloid negative (*SUVR* < 1.1), red for amyloid positive (*SUVR* > 1.2), and green for intermediate values. Thick, solid lines represent 15 years of evolution for a few representative participants. The colors denote whether they are positive (Pos) or negative (Neg) and the SUVR increase rate (stable, slow, or fast). See the legend. **(A)** PCs one and four. These two PCs have the maximum separation between amyloid-positive and negative projections. **(B)** PCs two and five. These two PCs have the minimum separation between amyloid-positive and negative projections among the first five PCA projections. **(A)** PCA dimensions 4 vs. 1. High amyloid correlation. **(B)** PCA dimensions 5 vs. 2. Low amyloid correlation.

By inspecting the histograms of negative and positive amyloid scans on each PC, we identified two dimensions that differentiate low vs high amyloid and three that do not. Figure 4A shows the distribution of subjects' baseline scans in the two PCs correlated with amyloid levels, and Figure 4B shows two from the non-correlated ones. The general dynamics are illustrated with lines representing the predicted short-term progression. Long-term progression is illustrated in a few selected subjects representing different dynamics, from stable to fast amyloid accumulators. These groups were defined based on the observed SUVR change in the real data after 4 years of evolution.

The first observation is that the predicted progression moves mainly toward regions populated with high amyloid subjects (to the right and sometimes upwards). This is an important consistency check. The dynamics estimated from consecutive scans of individual subjects reflect changes in the scan features and do not inherently account for the overall population distribution of amyloid load in the latent space. However, they are expected to align locally with the trend of increasing amyloid load observed in the population. The second observation is that the stable subjects (orange) or slow progressors (blue) travel a shorter distance on the x-axis, suggesting that this dimension represents amyloid progression. Conversely, the trajectories on the right do not differentiate fast and slow progressors, being very similar for all subjects. Most likely, these dimensions encode aspects of the image related to age that change at the same rate for all subjects.

### 3.2 Amyloid PET progression simulation

For each of the selected sample cases, illustrated in Figure 4, we generated the PET image at baseline and after four years of evolution. The evolution is computed in the five-dimensional PCA space and projected back to the latent space.

**Figure 5:**
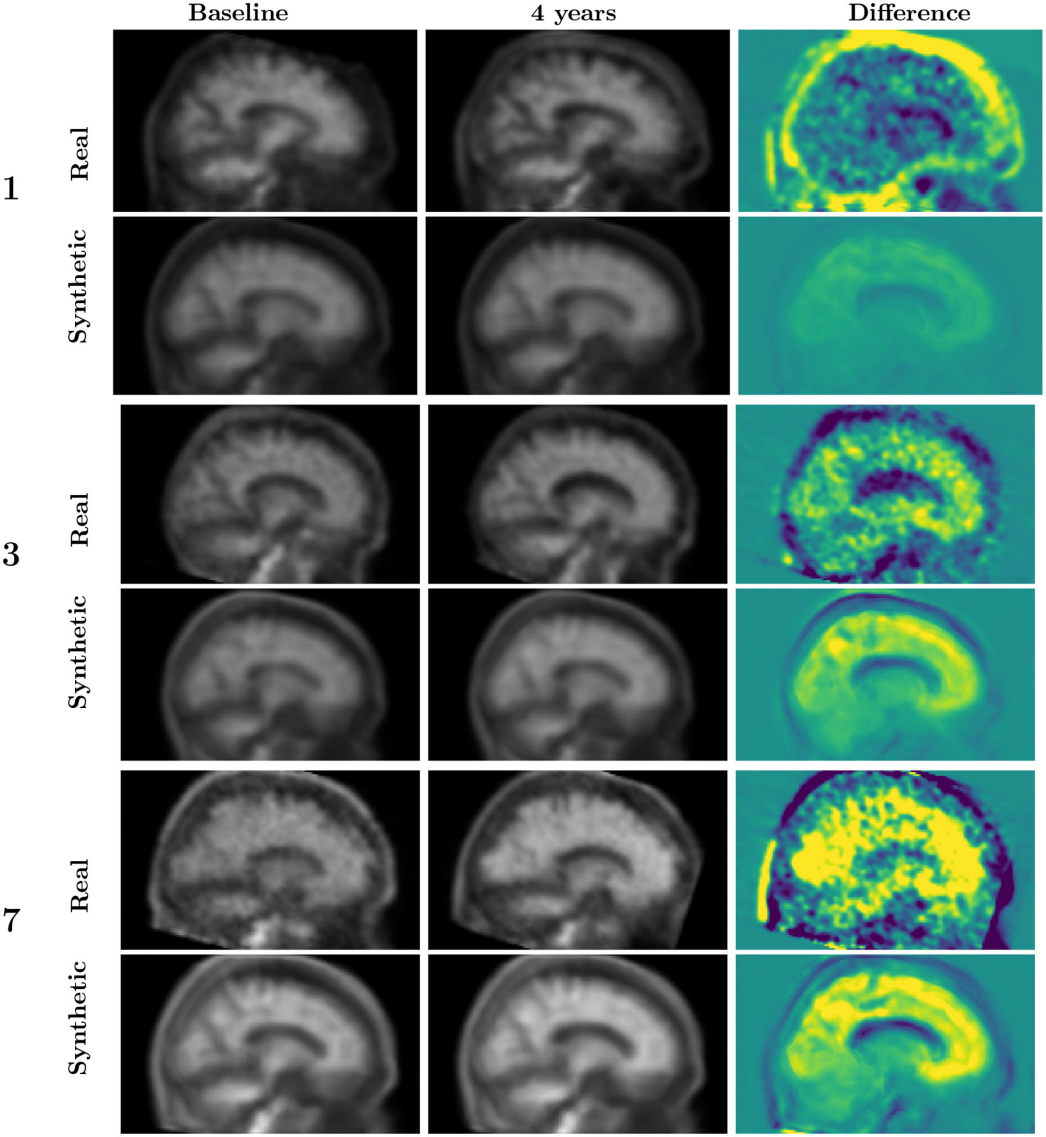
Synthetic vs. observed 4-year progression for participants experiencing brain amyloid accumulation. Participant 1 has a lower rate of SUVR increase than participand 3 and 7. The image range is from 0 to 4 for the gray images (first and second rows) and from −0.4 to 0.4 for the colored images (third rows), where blue corresponds to more negative values, yellow to positive values and green to zero. See Figure 4 for the corresponding latent representation.

**Figure 6:**
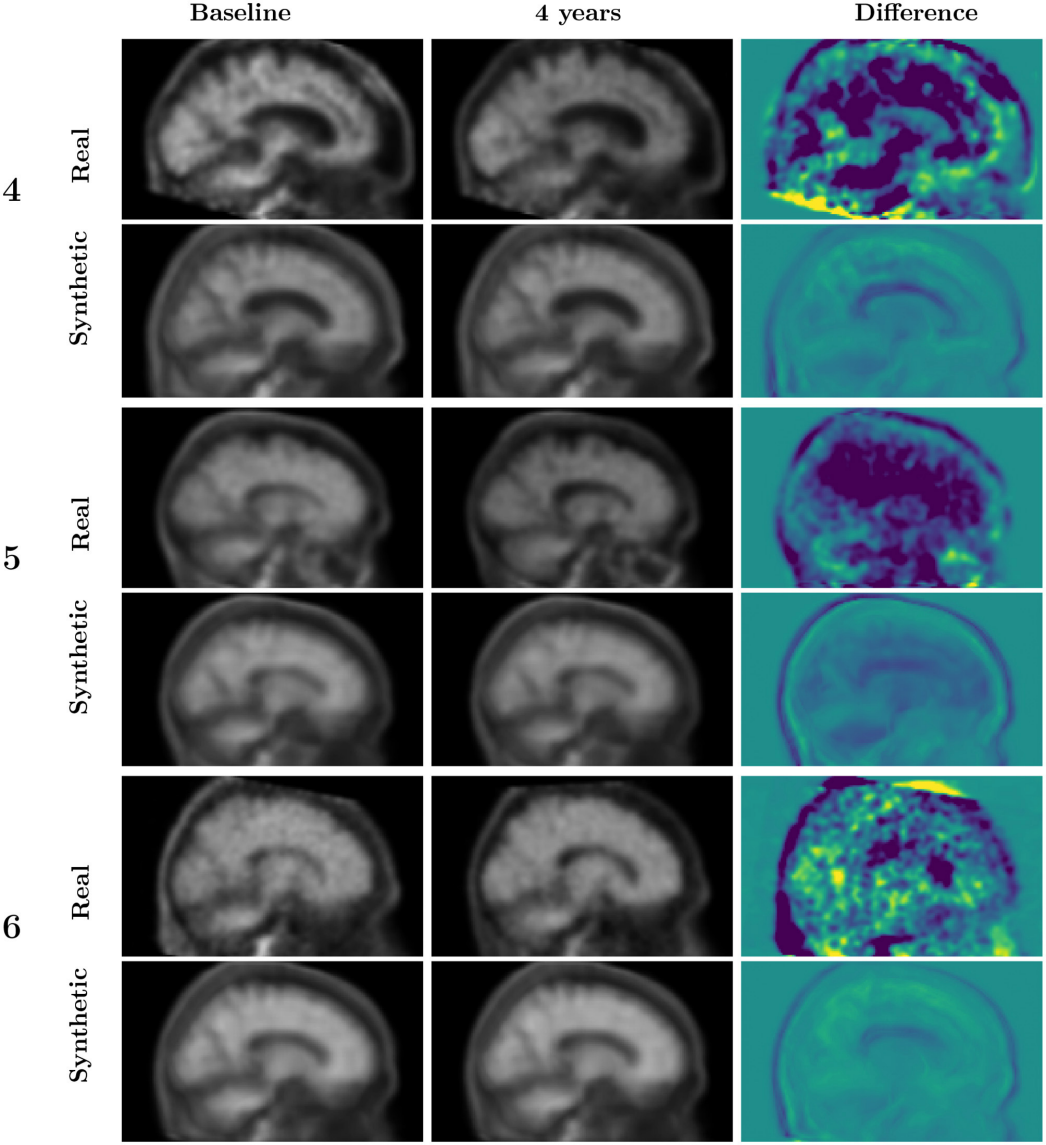
Synthetic vs. observed four-year progression for amyloid-positive and stable participants, i.e., those without significant brain amyloid accumulation as measured by SUVR change. See Figure 4 for the corresponding latent representation.

We generated a PET scan from the reconstructed latent vectors after projecting back from the PCA space to the latent representation space. Figure 5 shows the subject with slow Aβ rate of accumulation (labeled as 1 in Figure 4) and two subjects with a rapid Aβ accumulation (subjects numbered as 3 and 7). Figure 6 shows the images of the subjects who are not accumulating Aβ. The first row from both figures contains the baseline scans (original and reconstructed), the second row contains the image after 4 years (original and predicted), and the third row contains the difference.

The original and reconstructed images present similar patterns despite the latent representation being projected in a 5-dimensional space. Moreover, the predicted progress after four years was similar to the observed. In order to interpret these images, it is important to take into account that the Aβ accumulation is reflected in the ratio of the cortex signal to that of the cerebellum. The changes in amyloid load (either positive or negative) are not expected to occur outside the cortex. The patterns observed outside the cortex should be attributed to factors such as anatomical changes or misalignments. In the simulated images, the signal, when present, is almost exclusively concentrated on the cortex and ventricles. Signal change is expected to occur in ventricles because of the expansion they experience during age or dementia-related brain atrophy.

## 4 Discussion

Generative AI can address, in a single framework, various needs in medical neuroimaging modeling and analysis (Wang et al., 2023). These include progression modeling, statistical image analyses (such as statistical parametric mapping or voxel-level regression), AI interpretability [achieved through counterfactual analysis (Mertes et al., 2022)], and synthetic data simulation. This work aimed to explore the potential of generative AI for amyloid PET analysis and to illustrate that it can effectively achieve some of these goals with a concrete model architecture and a few practical applications.

Generative AI and GANs, in particular, have complex models that require non-naive optimization strategies. They have been intensively studied for 2D images until they could produce realistic images. Extension from 2D to 3D is not immediate, and memory requirements often make 3D deep neural networks models challenging (Singh et al., 2020; Volokitin et al., 2020). For example, the progression model for AD proposed in Bowles et al. (2018) was developed only for 2D images. For this reason, we have used 3D-StyleGAN (Hong et al., 2021) for image generation and representation. 3D-StyleGAN is a recently proposed model that leverages GANs for 3D brain image generation. It showed sufficient quality for 3D MRI image generation, including a latent space where we could do longitudinal analyses, and does not include unnecessary features for our purposes.

We extended the 3D-StyleGAN model with an encoder module. In the original 3D-StyleGAN model, image projection is obtained a posteriori via optimization (Hong et al., 2021). Our approach with an encoder module offers two significant benefits compared to the optimization approach. First, running a trained encoder is much faster. Second and more importantly, an encoder module allows for extending the architecture, using the latent representation of real images as input to other subnetworks or adding additional loss terms, facilitating further downstream tasks.

We showed that the latent representation encodes the information relative to amyloid load and can accurately predict global SUVR. This is not immediate, as the amyloid load is not directly reflected in the PET image intensities but should be derived from the relative intensities of the brain cortex to those of a reference region. Principal component analysis showed that this information is mainly encoded in the latent dimensions with higher variability in the population. Dimensionality reduction is particularly convenient for modeling dynamics because progression models are more robust in low-dimensional spaces, and it will become critical when extending the model with more biomarkers. Lastly, we illustrated that synthetic realistic-looking PET image trajectories could be generated.

Some previous works studying progression in neuroimaging did not provide a dynamic model describing rates of change or a continuous description of the progression path from healthy to diseased. In Yang et al. (2021), for example, progression pathways are inferred through a secondary analysis of the degree of expression or occupancy of a small set of identified patterns, and the progression is encoded in the order in which these patterns are visited. Other works proposed conditional networks to model progression (Zhao et al., 2021; Campello et al., 2022). These models predict next image from previous ones leveraging a U-Net backbone. They can predict image changes for a particular individual. However, they are not designed to generate completely synthetic image trajectories because the generator backbone can only generate images from other images and not from a latent vector alone.

Several limitations of the present study, as well as future research directions, can be highlighted. One major limitation is the lack of voxel-wise statistical analysis, which could provide a more detailed understanding of the spatial distribution of amyloid deposition. Both longitudinal and cross-sectional analysis (such as group comparison or regression) require the selection of a reference image. The analysis can be performed on the latent space, and statistical maps could be estimated with permutation tests (Bossa et al., 2010). Another limitation is the lack of quantitative evaluation of the predicted evolution, for example, comparing the SUVR of predicted trajectories with actual patient evolutions. Modeling the joint evolution of MRI and amyloid PET could be used to compute SUVR by traditional means to evaluate quantitatively the progression model. Furthermore, it would allow us to study the spatiotemporal SUVR distribution in predefined regions and compare it with previous works (e.g., Whittington et al., 2018). Modeling MRI is required to compute global or regional SUVR values because the precise extent of anatomical regions can not be determined from the PET image.

One potential extension of the present work is to evaluate the performance, benefit, and limitations of different generative approaches, including GANs, VAEs, and diffusion models (Bond-Taylor et al., 2022). Another promising extension is the joint modeling of PET amyloid with other dynamical variables, including imaging, biofluid biomarkers, or cognitive assessments. Aβ and tau levels in the blood or cerebrospinal fluid (CSF), neurodegeneration measured as brain atrophy in structural MRI, and metabolism (e.g., FDG PET) could inform more precisely the expected change in brain amyloid levels, and, in turn, amyloid PET could predict the expected progression on these biomarkers (Bossa et al., 2022; Bossa and Sahli, 2023).

This work contributes to recent efforts to build Digital Twins of the brain. The model's ability to provide a continuous, patient-specific prediction of disease progression can be used to tailor treatment plans to individual patients and inform clinical decisions (Ravi et al., 2022). Clinical trial designs for anti-amyloid treatments could also benefit from these models. Identifying the subjects with faster progression could reduce the costs associated with sample size or trial duration. Given the expected effect of a treatment on slowing amyloid accumulation, the model could be used to simulate PET images from the untreated group from specific populations and predict the effect size. Then, clinical trial costs could be optimized by tuning parameters such as follow-up duration (Bossa and Sahli, 2023). Some authors proposed using ODE-based progression models to simulate the effect of amyloid treatments on the disease course (Abi Nader et al., 2021). Such a causal model can be used to evaluate the treatment's efficacy by simulating the potential drug's effect on the synthetic images before conducting expensive and time-consuming clinical trials.

## Data availability statement

Publicly available datasets were analyzed in this study. This data can be found here: http://adni.loni.usc.edu.

## Ethics statement

The studies involving humans were approved by the Albany Medical Center Committee on Research Involving Human Subjects Institutional Review Board, the Boston University Medical Campus and Boston Medical Center Institutional Review Board, the Butler Hospital Institutional Review Board, the Cleveland Clinic Institutional Review Board, the Columbia University Medical Center Institutional Review Board, the Duke University Health System Institutional Review Board, the Emory Institutional Review Board, the Georgetown University Institutional Review Board, the Health Sciences Institutional Review Board, the Houston Methodist Institutional Review Board, the Howard University Office of Regulatory Research Compliance, the Icahn School of Medicine at Mount Sinai Program for the Protection of Human Subjects, the Indiana University Institutional Review Board, the Institutional Review Board of Baylor College of Medicine, the Jewish General Hospital Research Ethics Board, the Johns Hopkins Medicine Institutional Review Board, the Lifespan-Rhode Island Hospital Institutional Review Board, the Mayo Clinic Institutional Review Board, the Mount Sinai Medical Center Institutional Review Board, the Nathan Kline Institute for Psychiatric Research & Rockland Psychiatric Center Institutional Review Board, the New York University Langone Medical Center School of Medicine Institutional Review Board, Northwestern University Institutional Review Board, Oregon Health and Science University Institutional Review Board, the Partners Human Research Committee Research Ethics, the Board Sunnybrook Health Sciences Centre, the Roper St. Francis Healthcare Institutional Review Board, the Rush University Medical Center Institutional Review Board, the St. Joseph's Phoenix Institutional Review Board, the Stanford Institutional Review Board, the Ohio State University Institutional Review Board, the University Hospitals Cleveland Medical Center Institutional Review Board, the University of Alabama Office of the IRB, the University of British Columbia Research Ethics Board, the University of California Davis Institutional Review Board Administration, the University of California Los Angeles Office of the Human Research Protection Program, the University of California San Diego Human Research Protections Program, the University of California San Francisco Human Research Protection Program, the University of Iowa Institutional Review Board, the University of Kansas Medical Center Human Subjects Committee, the University of Kentucky Medical Institutional Review Board, the University of Michigan Medical School Institutional Review Board, the University of Pennsylvania Institutional Review Board, the University of Pittsburgh Institutional Review Board, the University of Rochester Research Subjects Review Board, the University of South Florida Institutional Review Board, the University of Southern, California Institutional Review Board, the UT Southwestern Institution Review Board, the VA Long Beach Healthcare System Institutional Review Board, the Vanderbilt University Medical Center Institutional Review Board, the Wake Forest School of Medicine Institutional Review Board, the Washington University School of Medicine Institutional Review Board, the Western Institutional Review Board, the Western University Health Sciences Research Ethics Board, and the Yale University Institutional Review Board. All methods were carried out in accordance with relevant guidelines and regulations. Further information about ADNI, including full study protocols, complete inclusion and exclusion criteria, and data collection and availability can be found at adni.loni.usc.edu. The studies were conducted in accordance with the local legislation and institutional requirements. Written informed consent for participation was not required from the participants or the participants' legal guardians/next of kin in accordance with the national legislation and institutional requirements. Written informed consent was obtained from the individual(s) for the publication of any potentially identifiable images or data included in this article.

## Author contributions

MB: Conceptualization, Formal analysis, Methodology, Supervision, Visualization, Writing – original draft, Writing – review & editing. AN: Data curation, Software, Writing – original draft, Writing – review & editing. AB: Methodology, Supervision, Writing – original draft, Writing – review & editing. HS: Conceptualization, Methodology, Supervision, Writing – original draft, Writing – review & editing.

## Footnotes

^1^ adni.loni.usc.edu

^2^ https://adni.loni.usc.edu/wp-content/uploads/2010/05/ADNI2_PET_Tech_Manual_0142011.pdf

^3^ https://adni.loni.usc.edu/methods/pet-analysis-method/pet-analysis/

^4^ https://www.fil.ion.ucl.ac.uk/spm/

^5^ https://surfer.nmr.mgh.harvard.edu/

^6^ https://adni.bitbucket.io/reference/docs/UCBERKELEYAV45/ADNI_AV45_Methods_JagustLab_06.25.15.pdf

## References

[B1] Abi Nader C. Ayache N. Frisoni G. B. Robert P. Lorenzi M. for the Alzheimer's Disease Neuroimaging Initiative (2021). Simulating the outcome of amyloid treatments in Alzheimer's disease from imaging and clinical data. Brain Commun. 3:fcab091. 10.1093/braincomms/fcab091 34085040 PMC8168944

[B2] Alamir M. Alghamdi M. (2022). The role of generative adversarial network in medical image analysis: an in-depth survey. ACM Comput. Surv. 55, 1–36. 10.1145/3527849

[B3] Arjovsky M. Chintala S. Bottou L. (2017). “Wasserstein generative adversarial networks,” in International Conference on Machine Learning (PMLR), 214–223.

[B4] Bond-Taylor S. Leach A. Long Y. Willcocks C. G. (2022). Deep generative modelling: a comparative review of VAEs, GANs, normalizing flows, energy-based and autoregressive models. IEEE Trans. Pattern Anal. Mach. Intell. 44, 7327–7347. 10.1109/TPAMI.2021.3116668 34591756

[B5] Bossa M. Berenguer A. D. Sahli H. (2022). “Non-parametric ODE-based disease progression model of brain biomarkers in Alzheimer's disease,” in Machine Learning in Clinical Neuroimaging, eds. A. Abdulkadir, D. R. Bathula, N. C. Dvornek, M. Habes, S. M. Kia, V. Kumar (Cham: Springer Nature Switzerland), 95–103. 10.1007/978-3-031-17899-3_1031168892

[B6] Bossa M. Zacur E. Olmos S. (2010). Tensor-based morphometry with stationary velocity field diffeomorphic registration: application to ADNI. Neuroimage 51, 956–969. 10.1016/j.neuroimage.2010.02.061 20211269 PMC3068621

[B7] Bossa M. N. Sahli H. (2023). A multidimensional ODE-based model of Alzheimer's disease progression. Sci. Rep. 13:3162. 10.1038/s41598-023-29383-5 36823416 PMC9950424

[B8] Bowles C. Gunn R. Hammers A. Rueckert D. (2018). “Modelling the progression of Alzheimer's disease in MRI using generative adversarial networks,” in Medical Imaging 2018: Image Processing, eds. E. D. Angelini, and B. A. Landman (International Society for Optics and Photonics, SPIE), 105741K. 10.1117/12.2293256

[B9] Campello V. M. Xia T. Liu X. Sanchez P. Martín-Isla C. Petersen S. E.. (2022). Cardiac aging synthesis from cross-sectional data with conditional generative adversarial networks. Front. Cardiov. Med. 9:983091. 10.3389/fcvm.2022.983091 36211555 PMC9537599

[B10] Chang J. Lee J. Ha A. Han Y. S. Bak E. Choi S.. (2021). Explaining the rationale of deep learning glaucoma decisions with adversarial examples. Ophthalmology 128, 78–88. 10.1016/j.ophtha.2020.06.036 32598951

[B11] Frid-Adar M. Diamant I. Klang E. Amitai M. Goldberger J. Greenspan H. (2018). GAN-based synthetic medical image augmentation for increased CNN performance in liver lesion classification. Neurocomputing 321, 321–331. 10.1016/j.neucom.2018.09.013

[B12] Gao X. Shi F. Shen D. Liu M. (2022). Task-induced pyramid and attention GAN for multimodal brain image imputation and classification in Alzheimer's disease. IEEE J. Biomed. Health Inform. 26, 36–43. 10.1109/JBHI.2021.3097721 34280112

[B13] Goodfellow I. J. Pouget-Abadie J. Mirza M. Xu B. Warde-Farley D. Ozair S.. (2014). “Generative adversarial nets,” in Proceedings of the 27th International Conference on Neural Information Processing Systems, NIPS'14 (Cambridge, MA, USA: MIT Press), 2672–2680.

[B14] Grothe M. J. Barthel H. Sepulcre J. Dyrba M. Sabri O. Teipel S. J.. (2017). In vivo staging of regional amyloid deposition. Neurology 89, 2031–2038. 10.1212/WNL.0000000000004643 29046362 PMC5711511

[B15] Hong S. Marinescu R. Dalca A. V. Bonkhoff A. K. Bretzner M. Rost N. S.. (2021). “3D-StyleGAN: a style-based generative adversarial network for generative modeling of three-dimensional medical images,” in Deep Generative Models, and Data Augmentation, Labelling, and Imperfections, eds. S. Engelhardt, I. Oksuz, D. Zhu, Y. Yuan, A. Mukhopadhyay, N. Heller, et al. (Cham: Springer International Publishing), 24–34. 10.1007/978-3-030-88210-5_3

[B16] Huang X. Belongie S. (2017). “Arbitrary style transfer in real-time with adaptive instance normalization,” in ICCV, 1510–1519. 10.1109/ICCV.2017.167

[B17] Jagust W. Landau S. Koeppe R. Reiman E. Chen K. Mathis C.. (2015). The Alzheimer's disease neuroimaging initiative 2 PET core: 2015. Alzheimer's Dement. 11, 757–771. 10.1016/j.jalz.2015.05.001 26194311 PMC4510459

[B18] Kang S. K. Kim D. Shin S. A. Kim Y. K. Choi H. Lee J. S. (2023). Fast and accurate amyloid brain PET quantification without MRI using deep neural networks. J. Nuclear Med. 64, 659–666. 10.2967/jnumed.122.264414 36328490 PMC10071781

[B19] Karras T. Laine S. Aila T. (2018). A style-based generator architecture for generative adversarial networks. CoRR, abs/1812.04948. 10.1109/CVPR.2019.00453 32012000

[B20] Karras T. Laine S. Aittala M. Hellsten J. Lehtinen J. Aila T. (2019). Analyzing and improving the image quality of StyleGAN. CoRR, abs/1912.04958. 10.1109/CVPR42600.2020.00813

[B21] Kim J.-Y. Suh H. Ryoo H. Oh D. Choi H. Paeng J. C.. (2019). Amyloid PET quantification via end-to-end training of a deep learning. Nucl. Med. Mol. Imag. 53, 340–348. 10.1007/s13139-019-00610-0 31723364 PMC6821901

[B22] Klunk W. E. Engler H. Nordberg A. Wang Y. Blomqvist G. Holt D. P.. (2004). Imaging brain amyloid in Alzheimer's disease with pittsburgh compound-B. Ann. Neurol. 55, 306–319. 10.1002/ana.20009 14991808

[B23] Kolinger G. Vállez García D. Willemsen A. Reesink F. Jong B. Dierckx R.. (2021). Amyloid burden quantification depends on PET and MR image processing methodology. PLoS ONE 16:e0248122. 10.1371/journal.pone.0248122 33667281 PMC7935288

[B24] Komori S. Cross D. J. Mills M. Ouchi Y. Nishizawa S. Okada H.. (2022). Deep-learning prediction of amyloid deposition from early-phase amyloid positron emission tomography imaging. Ann. Nucl. Med. 36, 913–921. 10.1007/s12149-022-01775-z 35913591

[B25] Koychev I. Hofer M. Friedman N. (2020). Correlation of Alzheimer disease neuropathologic staging with amyloid and tau scintigraphic imaging biomarkers. J. Nuclear Med. 61, 1413–1418. 10.2967/jnumed.119.230458 32764121

[B26] Lee J. Ha S. Kim R. E. Y. Lee M. Kim D. Lim H. K. (2022). Development of amyloid PET analysis pipeline using deep learning-based brain mri segmentation: a comparative validation study. Diagnostics 12:623. 10.3390/diagnostics12030623 35328176 PMC8947654

[B27] Maddury S. Desai K. (2023). DeepAD: a deep learning application for predicting amyloid standardized uptake value ratio through PET for Alzheimer's prognosis. Front. Artif. Intell. 6:1091506. 10.3389/frai.2023.1091506 36815006 PMC9939778

[B28] Mertes S. Huber T. Weitz K. Heimerl A. André E. (2022). Ganterfactual-counterfactual explanations for medical non-experts using generative adversarial learning. Front. Artif. Intell. 5:825565. 10.3389/frai.2022.825565 35464995 PMC9024220

[B29] Moffat G. Zhukovsky P. Coughlan G. Voineskos A. N. (2022). Unravelling the relationship between amyloid accumulation and brain network function in normal aging and very mild cognitive decline: a longitudinal analysis. Brain Commun. 4:fcac282. 10.1093/braincomms/fcac282 36415665 PMC9678202

[B30] Murray M. E. Lowe V. J. Graff-Radford N. R. Liesinger A. M. Cannon A. Przybelski S. A.. (2015). Clinicopathologic and 11C-Pittsburgh compound B implications of Thal amyloid phase across the Alzheimer's disease spectrum. Brain 138, 1370–1381. 10.1093/brain/awv050 25805643 PMC4407190

[B31] Pan Y. Liu M. Xia Y. Shen D. (2022). Disease-image-specific learning for diagnosis-oriented neuroimage synthesis with incomplete multi-modality data. IEEE Trans. Pattern Anal. Mach. Intell. 44, 6839–6853. 10.1109/TPAMI.2021.3091214 34156939 PMC9297233

[B32] Patow G. Stefanovski L. Ritter P. Deco G. Kobeleva X. for the Alzheimer's Disease Neuroimaging Initiative (2023). Whole-brain modeling of the differential influences of amyloid-beta and tau in Alzheimer's disease. Alzheimer's Res. Ther. 15:210. 10.1186/s13195-023-01349-9 38053164 PMC10696890

[B33] Pemberton H. G. Collij L. E. Heeman F. Bollack A. Shekari M. Salvadó G.. (2022). Quantification of amyloid PET for future clinical use: a state-of-the-art review. Eur. J. Nucl. Med. Mol. Imag. 49, 3508–3528. 10.1007/s00259-022-05784-y 35389071 PMC9308604

[B34] Ravi D. Blumberg S. B. Ingala S. Barkhof F. Alexander D. C. Oxtoby N. P. (2022). Degenerative adversarial neuroimage nets for brain scan simulations: application in ageing and dementia. Med. Image Anal. 75:102257. 10.1016/j.media.2021.102257 34731771 PMC8907865

[B35] Reith F. Koran M. E. Davidzon G. Zaharchuk G. (2020). Application of deep learning to predict standardized uptake value ratio and amyloid status on 18 F-Florbetapir PET using ADNI data. Am. J. Neuroradiol. 41, 980–986. 10.3174/ajnr.A6573 32499247 PMC7342760

[B36] Schön J. Selvan R. Petersen J. (2022). “Interpreting latent spaces of generative models for medical images using unsupervised methods,” in Deep Generative Models, eds. A. Mukhopadhyay, I. Oksuz, S. Engelhardt, D. Zhu, and Y. Yuan (Cham: Springer Nature Switzerland), 24–33. 10.1007/978-3-031-18576-2_329650303

[B37] Singh S. P. Wang L. Gupta S. Goli H. Padmanabhan P. Gulyás B. (2020). 3D deep learning on medical images: a review. Sensors 20:5097. 10.3390/s20185097 32906819 PMC7570704

[B38] Thal D. R. Rüb U. Orantes M. Braak H. (2002). Phases of Aβ-deposition in the human brain and its relevance for the development of AD. Neurology 58, 1791–1800. 10.1212/WNL.58.12.1791 12084879

[B39] Volokitin A. Erdil E. Karani N. Tezcan K. C. Chen X. Van Gool L.. (2020). “Modelling the distribution of 3D brain MRI using a 2D slice VAE,” in Medical Image Computing and Computer Assisted Intervention-MICCAI 2020, eds. A. L. Martel, P. Abolmaesumi, D. Stoyanov, D. Mateus, M. A. Zuluaga, S. K. Zhou (Cham: Springer International Publishing), 657–666. 10.1007/978-3-030-59728-3_64

[B40] Wang R. Bashyam V. Yang Z. Yu F. Tassopoulou V. Chintapalli S. S.. (2023). Applications of generative adversarial networks in neuroimaging and clinical neuroscience. NeuroImage 269:119898. 10.1016/j.neuroimage.2023.119898 36702211 PMC9992336

[B41] Whittington A. Sharp D. J. Gunn R. N. (2018). Spatiotemporal distribution of β-amyloid in Alzheimer disease is the result of heterogeneous regional carrying capacities. J. Nucl. Med. 59, 822–827. 10.2967/jnumed.117.194720 29146694 PMC5932528

[B42] Yang Z. Nasrallah I. M. Shou H. Wen J. Doshi J. Habes M.. (2021). A deep learning framework identifies dimensional representations of Alzheimer's disease from brain structure. Nat. Commun. 12:7065. 10.1038/s41467-021-26703-z 34862382 PMC8642554

[B43] Yi X. Walia E. Babyn P. (2019). Generative adversarial network in medical imaging: a review. Med. Image Anal. 58:101552. 10.1016/j.media.2019.101552 31521965

[B44] Zhang Z. Song Y. Qi H. (2017). “Age progression/regression by conditional adversarial autoencoder,” in 2017 IEEE Conference on Computer Vision and Pattern Recognition (CVPR) (Los Alamitos, CA, USA: IEEE Computer Society), 4352–4360. 10.1109/CVPR.2017.463

[B45] Zhao Y. Ma B. Jiang P. Zeng D. Wang X. Li S. (2021). Prediction of Alzheimer's disease progression with multi-information generative adversarial network. IEEE J. Biomed. Health Inform. 25, 711–719. 10.1109/JBHI.2020.3006925 32750952

